# Mass drug administration for soil-transmitted helminthiasis and schistosomiasis in selected districts of Rwanda: Coverage, implementation factors, and household water, sanitation, and hygiene conditions

**DOI:** 10.1371/journal.pntd.0014267

**Published:** 2026-05-07

**Authors:** Dieudonne Hakizimana, Ladislas Nshimiyimana, Jeanne Uwizeyimana, Jean Bosco Mbonigaba, Aimable Mbituyumuremyi, Nathan Hitiyaremye, Albert Tuyishime, Alison Ower, Karen Palacio, Tonya Huston, Ivy Sempele, Eugene Ruberanziza

**Affiliations:** 1 Department of Global Health, University of Washington, Seattle, Washington, United States of America; 2 Malaria and Other Parasitic Diseases, Rwanda Biomedical Center, Ministry of Health, Kigali, Rwanda; 3 Heart and Sole Africa, Kigali, Rwanda; 4 Institute of HIV/AIDS Disease Prevention and Control, Rwanda Biomedical Center, Ministry of Health, Kigali, Rwanda; 5 Programs, The END Fund, New York, United States of America; University of Buea, CAMEROON

## Abstract

**Background:**

In Rwanda, about 1 in 3 people are affected by soil-transmitted helminthiases (38.7%) and 1 in 4 by schistosomiasis in high-risk areas (27.2% by point-of-care circulating cathodic antigen), both associated with poor water, sanitation, and hygiene (WASH). Mass drug administration (MDA) is the primary control strategy, but reported coverage may not reflect true reach. This study assessed MDA coverage of albendazole/mebendazole and praziquantel in selected districts, identified reasons for non-reach, examined factors associated with uptake, and described household WASH conditions.

**Methods:**

A cross-sectional, community-based survey was conducted in five purposively selected districts using a stratified cluster design. Survey-weighted estimates summarized treatment reach (offered the drug), uptake (swallowed the drug), and household WASH conditions. Survey-weighted logistic regression was used to identify individual and implementation factors associated with uptake.

**Results:**

Albendazole/mebendazole uptake was 91.9% (95% CI: 84.3–96.0), closely matching reported coverage of 96%. The main reasons for non-reach were drug stockouts (23.7%), absence during MDA (15.8%), and unwillingness to take the drug (15.2%). Praziquantel uptake was 88.0% (95% CI: 78.7–93.6), consistent with reported coverage of 80%. For praziquantel, unwillingness to take tablets (29.6%) and absence during MDA (14.4%) were the most common reasons for non-reach.

Receiving sufficient information about MDA to make an informed decision was associated with higher odds of uptake for both ALB/MBZ (adjusted odds ratios [aOR]: 5.17, 95% CI: 2.01–13.27) and PZQ (aOR: 3.58, 95% CI: 1.33–9.64). For ALB/MBZ, finding MDA participation easy (aOR: 11.41, 95% CI: 2.59–50.16), feeling comfortable with the MDA distributor (aOR: 10.05, 95% CI: 2.43–41.47), and feeling comfortable with the MDA location (aOR: 3.23, 95% CI: 1.25–8.39) were each independently associated with higher uptake. For PZQ, males had significantly higher odds of uptake compared to females (aOR: 2.64, 95% CI: 1.15–6.07).

Among households, 65.6% used improved drinking water sources, 91.4% obtained water from public places, 50.7% treated their water, 84.4% had improved toilets, 51.6% had visibly clean toilets, and 62% lacked a handwashing station.

**Conclusion:**

MDA coverage in Rwanda exceeded WHO targets and closely matched reported estimates, reflecting strong implementation. Addressing remaining gaps in drug supply, MDA communication, and WASH infrastructure will be important to sustain and strengthen control of soil-transmitted helminthiases and schistosomiasis.

## Author summary

Soil-transmitted helminthiases (STH) and schistosomiasis (SCH) are parasitic infections that affect millions of people worldwide, especially in areas with poor access to water, sanitation, and hygiene (WASH) education. In Rwanda, these diseases are mainly addressed through nationwide treatment campaigns, where health workers give preventive tablets free of charge to all people aged 1 year and above. National reports often show that many people are reached, but it is important to check whether this information is accurate and to understand why some people might miss out.

In this study, we visited households in selected districts with reported high and moderate coverage to find out how many people were offered and actually swallowed the medicines for STH and SCH. We found that overall coverage was high and matched what the national program reported. People were more likely to take the medicine when they perceived that they had received sufficient information about the treatment campaign to make an informed decision, felt participation was easy, and felt comfortable with the distributor and the distribution location.

We also looked at household water, sanitation, and hygiene (WASH) conditions. While most families had improved toilets and safe water sources, many still lacked handwashing facilities and clean toilets. To keep making progress, it will be important to raise community awareness about national treatment campaigns, ensure treatment is delivered in a convenient and reliable way, and continue strengthening WASH services. Together, these steps can help control—and eventually eliminate—these diseases.

## Introduction

Despite substantial investment, soil-transmitted helminthiasis (STH) and schistosomiasis (SCH) remain major public health burdens in many low- and middle-income countries, including Rwanda. Globally, STH caused by *Ascaris lumbricoides*, *Trichuris trichiura*, and hookworms affects over 1.5 billion people, while SCH caused by *Schistosoma* species affects more than 250 million, 90% of whom live in Africa [1,2]. Both infections contribute to anemia, undernutrition, and impaired cognitive development, particularly in children, hindering progress in health, education, and economic development [3–6].

To address this burden, Rwanda’s Neglected Tropical Diseases (NTD) Program has implemented preventive chemotherapy through mass drug administration (MDA) since 2008, primarily delivered during biannual Maternal and Child Health (MCH) Weeks. Albendazole or mebendazole (ALB/MBZ) is used for STH, with praziquantel (PZQ) added where SCH prevalence exceeds 10% [7]. These efforts reduced STH prevalence among school-aged children (SAC) from 65.8% in 2007–2008 to 38.8% in 2020, with prevalence of 30.2% among preschool-aged children (pre-SAC), but rates remain high among adults (46.1%) [8]. For SCH, prevalence in children has declined, but more sensitive diagnostics suggest that transmission persists [7].

Rwanda has consistently reported high administrative coverage for MDA, often above 90% [7]. For example, an MDA round conducted from January 13–17, 2025, reported coverage of over 90% for ALB/MBZ among children, 90% among adults, and PZQ coverage of 88% for children and 84% for adults in selected districts. However, administrative data may overestimate true coverage because of reporting errors, inaccurate target population estimates, or overlooked groups, which should sustain pockets of transmission and delay elimination goals [9,10]. Population-based surveys provide a more reliable assessment of reach and uptake, can validate whether programs meet the World Health Organization (WHO) 75% effective coverage target, and help identify reasons for non-compliance and detect underserved populations and barriers to participation [11].

Household water, sanitation, and hygiene (WASH) are also critical for controlling STH and SCH. While MDA reduces infection intensity, it may not interrupt transmission without improved access to safe water and sanitation [12]. Rwanda has expanded WASH services, with access to improved water sources increasing from 73.8% in 2014 to 80.4% in 2020, though sanitation gains have been modest, rising only from 71.2% to 72.2% [13,14]. Describing WASH alongside MDA coverage can strengthen integrated approaches to elimination.

In this context, we conducted a population-based survey in selected districts of Rwanda to assess MDA coverage — including reach and drug uptake — for ALB/MBZ and PZQ, identify reasons for non-reach, examine individual and implementation factors associated with uptake, and describe household WASH conditions. These findings aim to inform program improvements to support Rwanda’s efforts to eliminate STH and SCH as public health problems.

## Methods

### Ethics statement

The study protocol was reviewed and approved by the Rwanda National Ethics Committee (Approval letter No. RNEC768/2024). Written informed consent was obtained from participants aged 18 years and above. For all participating children (<18 years), written informed consent was obtained from a parent or guardian. In addition, written assent was obtained from children aged 10–17 years. All participants, or their legal guardians for those under 18 years, were informed that participation was voluntary and that they could withdraw at any time without consequences.

### Study design

This study was a cross-sectional survey conducted in five districts in Rwanda, following the WHO recommended methodology for evaluating and validating MDA reported coverage [11].

### Study setting

The study was conducted in Rwanda, an East African country covering 26,338 square kilometers with an estimated population of 13.8 million in 2024, resulting in a population density of approximately 501 people per square kilometer [15,16]. The majority of the population (72.1%) resides in rural areas, and 69% are engaged in agriculture [15]. Administratively, Rwanda is divided into five provinces (North, East, West, South, and the City of Kigali), which are further subdivided into 30 districts, 416 sectors, 2,148 cells, and 14,837 villages, the smallest administrative unit [17]. The health system is organized hierarchically, starting at the community level (led by community health workers [CHWs]), followed by health centers, district hospitals, provincial hospitals and referral and specialized hospitals [18,19].

### Description of the MDA implementation approach and the target population in Rwanda

Recent surveys in Rwanda estimate national STH prevalence at 38.7% (95% CI: 37.9–39.4), highest among adults (46.1%), followed by SAC (39%) and pre-SAC (30%). *Ascaris lumbricoides* is most common (27%), followed by *Trichuris trichiura* (11.6%) and hookworm (10.7%). Most infections are light, although 8.1% of *A. lumbricoides* infections are moderate-to-heavy, while moderate-to-heavy hookworm and *T. trichiura* infections are rare (<1%). National SCH prevalence is 1.7% by Kato-Katz (95% CI: 1.5–1.9) and 27.2% (95% CI: 26.5–27.9) by point-of-care circulating cathodic antigen (POC-CCA) when trace positives are included. By POC-CCA, SCH is highest among pre-SAC (35.4%), followed by SAC (25.9%) and adults (20.9%). No *S. haematobium* infections have been detected, and moderate-to-heavy *S. mansoni* infections by Kato-Katz remain low (0.4%) [8].

For MDA, the implementation unit (IU) is the district for STH and the administrative cell for SCH. All 30 districts in Rwanda are endemic for STH, and MDA is implemented nationwide. Since 2021, following national STH/SCH remapping, the NTD program has, for the first time at the national scale, included adults (≥16 years) in STH MDA. Currently, two community- and school-based MDA rounds are conducted annually with ALB/MBZ, targeting pre-SAC (1–4 years), SAC (5–15 years) in all 30 districts, and adults in 25 districts. PZQ is administered annually in 1,013 administrative cells across 307 of Rwanda’s 416 sectors, targeting SAC and adults [7,8].

### Participants

The STH MDA coverage survey targeted individuals aged one year and older: specifically pre-SAC (1–4 years), SAC (5–15 years), and adults (≥16 years). The SCH coverage evaluation targeted individuals aged five years and older, including adults in endemic cells. All eligible household members in selected households were included. Pregnant women in the first trimester were excluded.

### Study size

The sample size was estimated using the WHO recommended survey sample builder for MDA coverage evaluations, which suggests a 30-cluster design (with villages serving as clusters in this study) [11,20]. The total sample was calculated assuming 50% coverage (to maximize the sample size for subgroup analyses), a 95% confidence level, a 5% margin of error, a design effect of 4 to account for intra-cluster correlation, and an expected non-response rate of 15%. This resulted in a target of 1,808 individuals. Based on a total fertility rate of 3.6 [15], the average household size was conservatively rounded down to 3 to maximize the number of households sampled. This translated to approximately 603 households in total. With a total of 30 villages (clusters), the survey was designed to include 20 households per village. Villages were distributed equally across the five districts, with six villages selected per district. The sample size was powered to estimate overall coverage across the study districts, not for district-level estimation.

### Sampling procedure

**Fig 1:**
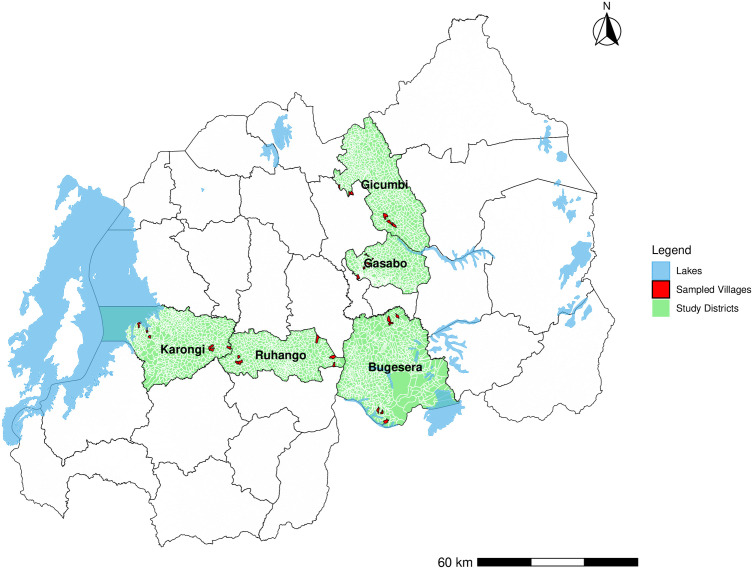
Map of study districts with selected villages highlighted in red.

Five districts were purposively selected from Rwanda’s 30 districts (the implementation unit for STH) to represent diverse implementation settings for STH and SCH (Fig 1). Selection was based on known or suspected coverage challenges, endemicity levels, and the need to better understand MDA delivery for both diseases. One district was selected from each province and from the City of Kigali: Karongi (Western — low ALB/MBZ and PZQ coverage, many SCH-endemic cells); Gicumbi (Northern — high ALB/MBZ and PZQ coverage, many SCH-endemic cells); Bugesera (Eastern — high ALB/MBZ coverage); Ruhango (Southern — high ALB/MBZ coverage); and Gasabo (Kigali — low ALB/MBZ and PZQ coverage, many SCH-endemic cells).

The map was created by the authors using Rwanda administrative boundary shapefiles obtained during a previous project. The same shapefiles are publicly available through the National Institute of Statistics of Rwanda (NISR) ArcGIS Hub: https://hub.arcgis.com/search?q=rwanda&tags=NISR

Sampling was conducted in R (version 4.5) using a fixed random seed of 2 to ensure reproducibility. The sampling frame included all villages in the selected districts, with information on district, sector, cell, and PZQ eligibility status. Sampling at each stage was conducted without replacement.

A stratified two-stage sampling strategy was applied. In each district, two sectors were randomly selected in the first stage to support logistical efficiency and avoid excessive travel between villages. For Karongi, Gicumbi, Bugesera, and Ruhango districts, three villages were randomly selected within each sector, resulting in six villages per district. PZQ coverage assessment was limited to selected PZQ-eligible villages in Karongi, Gicumbi, and Gasabo. To ensure adequate representation of PZQ-eligible areas for SCH, each district’s village sample was checked to confirm that at least two PZQ-eligible villages were included per district. If this minimum criterion was not met, additional PZQ-eligible villages not already selected were randomly sampled and added; however, all districts reached the target in the initial draw.

In Gasabo district, which is more urban with specific rural areas eligible for PZQ, a slightly different stratified approach was used. After selecting two sectors, villages were stratified by PZQ eligibility based on endemicity mapping. Five PZQ-eligible villages were randomly selected to ensure sufficient clusters for PZQ coverage estimation, and one PZQ-ineligible village was selected to contribute to ALB/MBZ coverage estimation.

In each selected village, the Community and Environmental Health Officer of the local health center worked with CHWs to compile household lists. Households were grouped into segments based on informal local administrative clusters (*isibo*, typically 20–50 households) to ensure segments contained approximately 50–60 households each. One segment was randomly selected per village. Within each segment, 20 households were randomly selected for the survey, except in the five SCH-eligible villages in Gasabo district, where 24 households were selected to meet the required PZQ sample size. All eligible individuals within selected households were invited to participate.

### Data sources and measurement

#### Data collection tool.

Data were collected using a structured household questionnaire designed and programmed in REDCap. The questionnaire included four modules. The first module covered household location and identification, as well as demographic characteristics such as sex and age.

The second module captured information on whether eligible individuals were offered and took ALB/MBZ (for those aged one year and above) and PZQ (for those aged five years and above). For each drug, respondents were asked about reasons the drug was not offered (e.g., underage, sick, absent, did not hear about the MDA, drug ran out, no distributor came, tablets purchased privately, unwillingness to receive, other reasons) and reasons the drug was offered but not swallowed (e.g., fear of side effects, bad taste, not feeling sick, insufficient information, other). The questionnaire also captured reasons for swallowing the offered drug (e.g., to treat disease, because it was given for free, or because of advice from a CHWs, etc.). These questions were adapted from the Coalition for Operational Research on Neglected Tropical Diseases (COR-NTD) coverage survey tools [20].

The third module collected information related to MDA implementation. This section included questions on participants’ experiences and perceptions of the drug distribution, such as awareness of the MDA in advance, source of information about the MDA, and what participants liked or disliked about the MDA. The survey also asked whether participants preferred the distribution mode used or would prefer an alternative. MDA acceptability was explored through questions about whether participants felt they had enough information to decide whether to take the drugs, perceived waiting time, satisfaction with and trust in distributors, comfort with the MDA location, perceptions of MDA importance compared to other child health interventions, whether participation was perceived as easy, and willingness to accept MDA drugs again in the future. In addition to insights from previous coverage surveys, these questions were adapted from the DeWorm3 coverage questionnaire [21].

The fourth module collected household WASH information. This module asked the head of household about their main source of drinking water, the type of water source used, and its location (on premises or outside the dwelling). The questionnaire also collected data on whether the household treated drinking water and which treatment methods were used. Sanitation questions covered the type of toilet facility used, its location (inside or outside the dwelling), and whether the facility was shared with other households. To understand hygiene conditions, enumerators observed the cleanliness of the toilet, checked whether a handwashing facility was present on the premises, and noted whether soap and water were available at the time of the visit. The WASH questions were adapted from the World Health Organization (WHO)/United Nations Children’s Fund (UNICEF) Joint Monitoring Programme (JMP) for WASH [22].

The questionnaire was developed in English and translated into Kinyarwanda (the local language) by bilingual members of the study team. It was pilot-tested before use to ensure clarity and cultural appropriateness for data collectors and respondents.

#### Recruitment and data collection procedures.

Data collection was conducted in person by experienced data collectors who had previously participated in MDA coverage surveys and were fluent in both English and Kinyarwanda. All data collectors completed a one-day refresher training, which covered the survey objectives, use of the electronic questionnaire, and ethical principles, including obtaining informed consent and maintaining confidentiality. Data collection took place from 10 April to 24 April 2025.

Households were randomly selected from household lists in each segment and each data collector was assigned specific households and worked with CHWs to locate them. The questionnaire was administered in Kinyarwanda using electronic tablets to ensure comprehension and accuracy. Data collectors conducted structured interviews by reading each question aloud and recording responses directly into the digital tool. Questions about whether the drug was offered and swallowed were answered directly by participants aged over 10 years; for children aged 10 years and younger, a guardian provided the responses. If no eligible members were present or if the household was absent and not expected to return later that day, the data collector proceeded to the next household; no replacements were made, as the sample size accounted for non-response. However, if a respondent (older than 10 years) was temporarily absent but expected to return later the same day, the household was revisited.

The field supervisors reviewed data daily and followed up with data collectors to resolve any issues while they were still in the field. All completed data were synchronized daily to a secure server hosted by the Rwanda Biomedical Centre, which complies with national data protection regulations.

#### Bias and mitigation measures.

To minimize potential sources of bias, several steps were implemented throughout the study design and data collection process. To strengthen validity, most survey questions were adapted from recommended tools to assess MDA coverage and had been previously used in low-income settings [20–22]. The household questionnaire was developed in English and translated into Kinyarwanda by bilingual study team members to ensure linguistic and cultural appropriateness. The tool was pilot-tested before use to confirm that all questions were clear and easily understood by participants, reducing the risk of misinterpretation or response error.

Data collection was conducted by trained and experienced data collectors who were independent of the MDA implementation teams to reduce interviewer bias and conflicts of interest. Although CHWs assisted data collectors in locating selected households, they did not remain present during the interviews to limit the potential for social desirability bias, since CHWs are directly involved in delivering MDA.

Data were collected electronically using REDCap, which included built-in logic and range checks to minimize data entry errors and missing responses. In addition, daily quality control checks were conducted by field supervisors, who provided feedback to data collectors so that any discrepancies could be addressed promptly in the field. Finally, data cleaning and analysis were carried out independently by the principal investigator, who was not affiliated with the NTD program implementing the MDA.

### Variables

#### Dependent variables.

The primary dependent variables were reach and swallow *(*survey coverage). Reach was defined as whether an eligible participant, or the guardian for children under 10 years, self-reported being offered ALB/MBZ for STH or PZQ for SCH during the most recent MDA round, including presenting at a fixed distribution site or being contacted by a community health worker during house-to-house distribution where applicable. Swallow was defined as whether the participant, or the guardian for children under 10 years, reported that the offered drug(s) were actually swallowed, serving as the measure of survey-based coverage. In addition, information was collected on the reasons why the drug was not offered, why it was swallowed, or why it was not swallowed.

#### Independent variables.

Details of all independent variable questions and response options are described in the data collection tool section. Specifically, the independent variables included the following:

**Sociodemographic variables:** These included district of residence, age group, and sex. Age was categorized as pre-SAC (1–4 years), SAC (5–15 years), and adults aged 16 years and older. Sex was recorded as male or female.

**MDA-related implementation variables:** These included participants’ awareness of the MDA campaign in advance, their sources of information (with the option to report more than one), and their experiences and perceptions of how drug distribution was conducted**.** Variables included whether participants liked or disliked aspects of the MDA, felt they had enough information to decide whether to take the drugs, whether waiting time was appropriate, trust and satisfaction with distributors, comfort with the distribution location, and willingness to participate in future MDA rounds. For each household-level perception variable, a binary indicator was created by assigning a value of 1 when at least one household member reported a positive response (i.e., “Yes,” “Agree,” or “Strongly agree”), and 0 when no such response was recorded within the household.

**Household WASH variables:** These described household water, sanitation, and hygiene conditions relevant to STH and SCH transmission risk. Drinking water source and type of toilet were categorized based on WHO/UNICEF JMP standards for WASH [22]. A drinking water source was classified as improved if it included piped water into the dwelling, yard/plot, or a public tap; protected dug wells; boreholes or tubewells; protected springs; rainwater; bottled water; or water from tanker trucks. Toilet facilities were classified as improved if they included flush toilets connected to sewer systems, septic tanks, or pit latrines; pit latrines with a slab; twin pit latrines with a slab; composting toilets, or flush toilets with an unknown destination if the technology was otherwise improved [22]. Toilets were further categorized by whether they were shared with other households, and handwashing was defined by the presence of a handwashing facility with soap and water.

### Statistical methods

All survey data were downloaded in Microsoft Excel format and exported to R version 4.5 for cleaning and analysis. The dataset was checked for duplicates, inconsistencies, and missing values before analysis. Descriptive statistics were generated using frequencies and percentages to summarize categorical variables, including participants’ sociodemographic characteristics, MDA implementation indicators, WASH variables, and outcome variables.

Sampling weights reflected the multi-stage design and were adjusted for oversampling of villages to ensure adequate representation of PZQ-eligible areas. Analyses incorporated these weights using the survey package in R, and confidence intervals (CIs) for proportions were calculated with the logit method [23].

Survey-weighted univariable and multivariable logistic regression models were fitted for ALB/MBZ and PZQ separately to identify factors associated with drug uptake. Models included district, sex, and household-level MDA implementation variables as covariates. Prior to multivariable model fitting, we examined potential collinearity among covariates through pairwise correlations and generalized variance inflation factors (GVIF). Where strong collinearity was detected (|r| > 0.7), we retained the variable with the greatest conceptual relevance to drug uptake based on the literature and experience from previous chemotherapy campaigns. Covariates in the final models demonstrated acceptable levels of collinearity, with GVIF^(1/(2·Df)) values below 5 for all predictors. Statistical significance was defined as p < 0.05, and odds ratios (ORs) and adjusted odds ratios (aORs) with 95% CIs are reported; univariable results are provided in S1 Appendix.

To validate reported coverage estimates, we calculated the average reported coverage across the study districts and compared it with the survey-based coverage estimate (drug uptake). Reported coverage was classified as underestimated if it was below the lower bound of the survey estimate’s 95% CI, validated if it fell within the CI, or overestimated if it exceeded the upper bound [11].

## Results

### Sociodemographics characteristics of study participants

Table 1 shows the sociodemographic characteristics of study participants. A total of 3,010 individuals were included in the ALB/MBZ coverage analysis and 908 in the PZQ analysis. For ALB/MBZ, district proportions ranged from 17.6% in Gicumbi to 22.2% in Bugesera; for PZQ, Gasabo accounted for 46.9% of participants and Karongi for 26.1%. Among ALB/MBZ participants, 49.7% were adults (≥16 years), 31.2% were SAC (5–15 years), and 19.1% were pre-SAC (1–4 years). For PZQ, 68.2% were adults and 31.8% were SAC; no pre-SAC were included. Females comprised 52.7% of ALB/MBZ participants and 54.4% of PZQ participants.

**Table 1:** Sociodemographic characteristics of study participants.

Variable	Categories	Albendazole/Mebendazole N = 3010		PZQ N = 908	
		n	%	n	%
District	Bugesera	668	22.2	–	–
	Gasabo	637	21.2	426	46.9
	Gicumbi	530	17.6	245	27
	Karongi	581	19.3	237	26.1
	Ruhango	594	19.7	–	–
Age categories	pre-SAC (1–4)	576	19.1	–	–
	SAC (5–15)	939	31.2	289	31.8
	Adults (16+)	1494	49.7	619	68.2
Sex	Female	1585	52.7	493	54.4
	Male	1423	47.3	413	45.6

### Participants’ experiences and perceptions of MDA implementation

Variables related to the implementation of MDA activities are presented in Table 2. Overall, 85.2% of participants were aware of the drug distribution in advance. Among those aware, the main sources of information were CHWs or teachers (69.2%), town criers (42.1%), and radio jingles (27.1%). Other sources, such as brochures, banners, or social media, were less common.

**Table 2:** Variables related to the implementation of MDA activities.

Variables	Categories	%	95% CI
Aware of drug distribution beforehand		85.2	[80 – 89.2]
Source of information about MDA (among those who were aware beforehand)	CHWs or teacher	69.2	[53.3 – 81.6]
	Town criers	42.1	[18.5 – 69.9]
	Radio jingles	27.1	[19 – 37.1]
	Family/friend/neighbor	23.4	[10.9 – 43.4]
	Community/religious leader	13.1	[6.9 – 23.5]
	Ministry of health poster	8.4	[5.3 – 12.9]
	Radio talk show	5.8	[2 – 15.9]
	Health professional	4.5	[1 – 18.6]
	Social media	3	[1.1 – 8.4]
	Television	2.8	[0.2 – 26.3]
	Brochures/flyers/ Banners	2.3	[0.3 – 13.9]
What the participant liked about the MDA implementation	Free drugs	55	[45.2 – 64.5]
	Trusted CHWs	44.6	[34.1 – 55.7]
	No long wait for drugs	43.9	[26.2 – 63.2]
	House-to-house treatment	33.8	[14.5 – 60.6]
	Other	7.2	[3.5 – 14.6]
	Nothing in particular	2.6	[1.3 – 5]
What the participant disliked about the MDA implementation	Nothing in particular	78.9	[62.4 – 89.4]
	Adverse drug reactions	9.6	[2.9 – 27.2]
	Didn’t treat other diseases	7.4	[3.2 – 16.4]
	Other	4.8	[2.6 – 8.6]
	Inconvenient time	3.9	[0.4 – 28.3]
	Long waiting time	1.6	[0.2 – 11.3]
	Drugs unavailable/finished	1.2	[0.3 – 4.4]
	Unfriendly distributors	0.8	[0.1 – 5.5]
MDA is a useful intervention		99	[98.1 – 99.5]
Preferred mode of drug distribution during MDA	House-to-house delivery	72.8	[55.8 – 85]
	Distribution at schools	38.2	[18 – 63.3]
	Fixed point delivery	29.9	[22.8 – 38.1]
	Health center delivery	23	[11 – 41.9]
	Other	10.1	[6.8 – 14.6]
Aware of neighbors taking MDA		83.5	[72.8 – 90.5]
Received enough MDA information to decide		89.8	[85.2 – 93.1]
MDA did not take too much time		91.3	[76.8 – 97.1]
Found MDA participation easy		96.7	[95.3 – 97.7]
Would accept MDA drug again in the future		98	[96.9 – 98.7]
Comfortable with MDA location		96.6	[95 – 97.7]
Comfortable with MDA distributor		96.2	[92.6 – 98.1]
Comfortable with mixed drug intake		96.9	[94.7 – 98.2]
MDA importance vs other child interventions	Most important	87.8	[77.4 – 93.8]
	Moderately important	10.7	[5 – 21.4]
	Least important	1.5	[0.7 – 3.2]

Participants most commonly liked aspects such as free drugs (55%), house-to-house treatment (33.8%), trusted CHWs (44.6%), and no long wait for drugs (43.9%). The majority (78.9%) reported nothing they disliked about the MDA. Nearly all participants considered MDA useful (99%). Most reported being comfortable with the location (96.6%), the distributors (96.2%), and taking drugs together (96.9%).

House-to-house delivery was the most preferred mode (72.8%), followed by school-based distribution (38.2%) and fixed-site delivery (29.9%). Most participants felt they received enough information to decide (89.8%), did not find the process time-consuming (91.3%), found participation easy (96.7%), and would accept MDA again in the future (98%). The majority were aware of neighbors participating in MDA (83.5%), and 87.8% viewed MDA as more important than other child health interventions.

### Coverage estimates, administrative comparison, and reasons for ALB/MBZ and PZQ reach and uptake

Table 3 shows the survey-based coverage estimates (reach and uptake) for ALB/MBZ and PZQ compared with reported administrative coverage. Overall, 92.5% (95% CI: 85.8–96.2) of surveyed participants reported being offered ALB/MBZ, and 91.9% (95% CI: 84.3–96.0) reported swallowing it. The average reported administrative coverage for ALB/MBZ across the five districts was 96%, which fell within the 95% CI of the survey estimate. For PZQ, survey-based reach and uptake were 88.7% (95% CI: 79.4–94.2) and 88.0% (95% CI: 78.7–93.6), respectively, compared to an average reported administrative coverage of 80% across the eligible districts, which also fell within the CI.

**Table 3:** Survey-based coverages estimates (reach and uptake) for ALB/MBZ and PZQ and comparison with administrative coverage by district.

District	Albendazole/Mebendazole				Praziquantel			
	Reported/ Administrative coverage		Survey estimates% [95% CI]		Reported/ Administrative coverage		Survey estimates% [95% CI]	
	%	Average	Reach	Drug uptake	%	Average	Reach	Drug uptake
Bugesera	97	96%	**92.5[85.8 – 96.2]**	**91.9[84.3 – 96]**	–	80%	**88.7[79.4 – 94.2]**	**88[78.7 – 93.6]**
Gasabo	93				72			
Gicumbi	97.7				90			
Karongi	92.3				78			
Ruhango	99.7				–			

**Fig 2:**
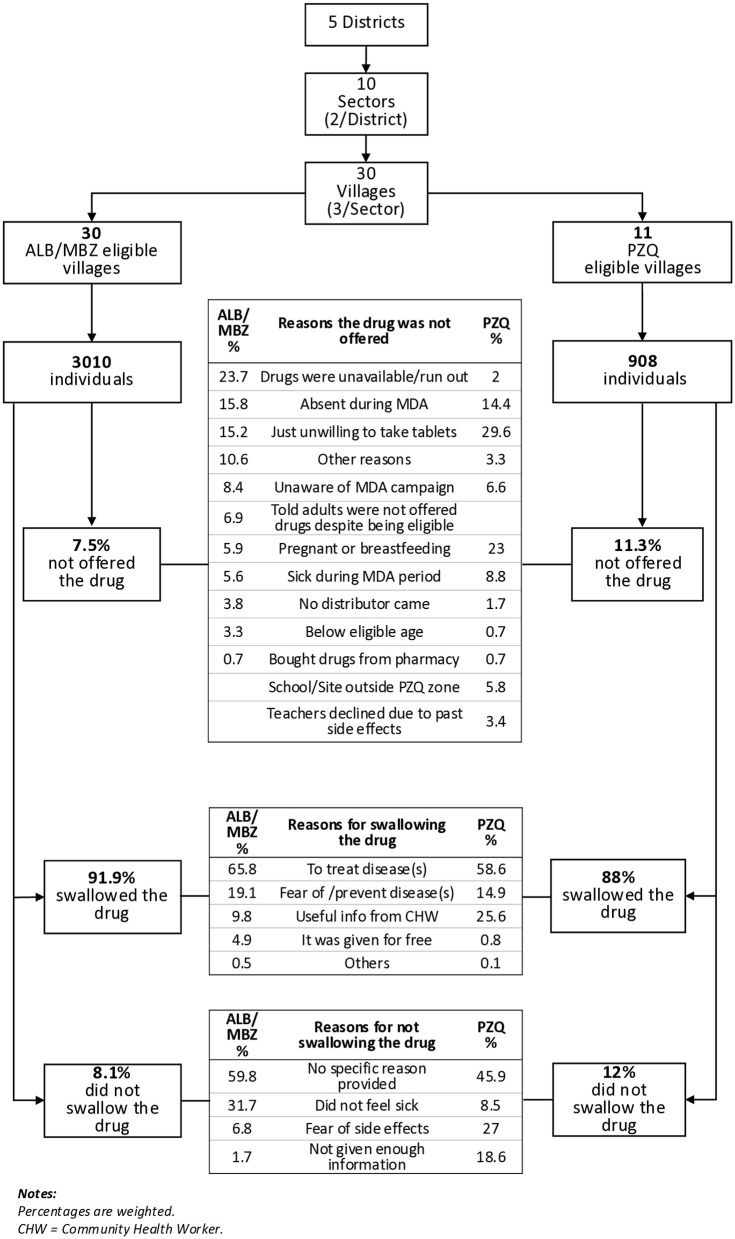
Sampling flow and reported reasons for drug offering and uptake of ALB/MBZ and PZQ during MDA.

Fig 2 presents the sampling flow, and the reasons participants gave for whether they were offered and swallowed ALB/MBZ and PZQ during the MDA. Overall, 7.5% of ALB/MBZ-eligible participants and 11.3% of PZQ-eligible participants reported not being offered the drug. The most common reasons for ALB/MBZ not being offered were drug unavailability or stockouts (23.7%), participant absence during MDA (15.8%) or unwillingness to take the drug (15.2%). For PZQ, the main reasons were unwillingness to take the tablets (29.6%), pregnancy or breastfeeding (23%), and absence during MDA (14.4%).

Among those who swallowed ALB/MBZ, the main reasons reported were to treat disease(s) (65.8%), to prevent disease(s) (19.1%), and receiving useful information from a CHW (9.8%). For PZQ, participants most commonly reported taking the drug to treat disease(s) (58.6%), based on information received from a CHW (25.6%), or to prevent disease(s) (14.9%).

Among the 8.1% who did not swallow ALB/MBZ, most did not provide any reason or did not want the drug (59.8%), followed by not feeling sick (31.7%) and fear of side effects (6.8%). For the 12% who did not swallow PZQ, the main reasons were no specific reason or unwillingness (45.9%), fear of side effects (27%), and not feeling sick (8.5%).

Table 4 shows that drug uptake was consistently high across sociodemographic groups for both ALB/MBZ and PZQ. ALB/MBZ uptake was similar by sex, estimated at 91.4% among females and 92.5% among males. PZQ uptake was likewise comparable by sex, at 86.0% among females and 90.4% among males. By age group, ALB/MBZ uptake was highest among pre-SAC (1–4 years) at 93.2%, followed by SAC (5–15 years) at 92.0%, and adults (16 years and older) at 91.4%. For PZQ, uptake was higher among SAC (92.8%) than among adults (85.8%). Survey-weighted estimates and unadjusted logistic regression results for both ALB/MBZ and PZQ reach and uptake by sociodemographic and implementation variables are provided in S1 Appendix.

**Table 4:** Weighted estimates of drug uptake by sociodemographic characteristics.

Variables	Categories	ALB/MBZ		PZQ	
		%	95% CI	%	95% CI
Sex	Female	91.4	[80.8 – 96.4]	86	[76.2 – 92.2]
	Male	92.5	[87.5 – 95.7]	90.4	[78.1 – 96.1]
Age categories	pre-SAC (1–4)	93.2	[81.6 – 97.7]	–	–
	SAC (5–15)	92	[72.7 – 98]	92.8	[81 – 97.5]
	Adults (16+)	91.4	[89.2 – 93.2]	85.8	[73.6 – 92.9]

### Factors associated with ALB/MBZ and PZQ uptake (survey-weighted multivariable logistic regression)

Table 5 presents survey-weighted multivariable logistic regression results for factors associated with drug uptake for ALB/MBZ and PZQ. After adjusting for other factors, individuals in households where at least one member perceived having received sufficient information about MDA to make an informed decision had higher odds of ALB/MBZ uptake (aOR: 5.17, 95% CI: 2.01–13.27; p = 0.001). Similarly, individuals in households where at least one member found MDA participation easy (aOR: 11.41, 95% CI: 2.59–50.16; p = 0.001), felt comfortable with the MDA distributor (aOR: 10.05, 95% CI: 2.43–41.47; p = 0.001), or felt comfortable with the MDA location (aOR: 3.23, 95% CI: 1.25–8.39; p = 0.016) were each independently associated with higher ALB/MBZ uptake.

**Table 5:** Factors associated with uptake of ALB/MBZ and PZQ: survey-weighted multivariable logistic regression results.

Variables	Category	ALB/MBZ			PZQ		
		aOR	95% CI	P value	aOR	95% CI	P value
District	Bugesera	Ref					
	Gasabo	0.23	0.1–0.52	<0.001	Ref		
	Gicumbi	0.42	0.2–0.86	0.018	0.66	0.12–3.72	0.634
	Karongi	0.81	0.2–3.23	0.763	0.95	0.2–4.41	0.946
	Ruhango	0.22	0.1–0.47	<0.001			
Sex	Female						
	Male	0.86	0.48–1.53	0.604	2.64	1.15–6.07	0.023
**Community perceptions variables (Ref: No)**							
Aware of drug distribution beforehand		1.55	0.97–2.47	0.066	2.45	0.71–8.39	0.154
Aware of neighbors taking MDA		1.65	0.95–2.89	0.078	1.57	0.63–3.9	0.336
Received enough MDA information to decide		5.17	2.01–13.27	0.001	3.58	1.33–9.64	0.012
MDA is a useful intervention		0.04	0–3.66	0.162	2.94	0.38–22.89	0.303
Found MDA participation easy		11.41	2.59–50.16	0.001	6.45	0.71–59.04	0.099
Comfortable with MDA distributor		10.05	2.43–41.47	0.001	1.22	0.43–3.5	0.710
Comfortable with MDA location		3.23	1.25–8.39	0.016	2.19	0.27–17.4	0.460

For PZQ, after adjusting for other factors, males had significantly higher odds of uptake compared to females (aOR: 2.64, 95% CI: 1.15–6.07; p = 0.023). In addition, individuals in households where at least one member perceived having received sufficient information about MDA to make an informed decision had higher odds of uptake (aOR: 3.58, 95% CI: 1.33–9.64; p = 0.012). Awareness of drug distribution beforehand, awareness of neighbors taking MDA, and perceived usefulness of MDA were not statistically significant predictors for either drug, nor were ease of participation, comfort with the distributor, and comfort with the MDA location for PZQ uptake.

### Household WASH conditions

Table 6 presents weighted estimates of household WASH indicators. Overall, 65.6% of households used an improved drinking water source. Most households collected drinking water from a public place (91.4%), and only 6.9% reported having a source on their premises. About half of households (51.7%) reported treating their drinking water, most commonly by boiling (82.1%). Among households that stored drinking water (66.2%), jerry cans were the most commonly used container (93.8%), and the majority (60%) cleaned their storage containers less than seven times per week.

**Table 6:** Weighted estimates of WASH-related indicators.

Variables	Categories	%	95% CI
Water source location	Public place	91.4	[65.1 – 98.4]
	Neighbor’s premise	1.8	[0.4 – 8]
	On premises	6.9	[1 – 33.9]
Drinking water source improved ^1^		65.6	[39.6 – 84.8]
Household treats drinking water		51.7	[42.5 – 60.8]
Water treatment methods among those who treat their drinking water	Boil	82.1	[52.9 – 94.9]
	Add bleach/chlorine	8.6	[3.6 – 18.9]
	Use water filter	9.2	[1.8 – 36]
	Solar disinfection	0.1	[0 – 1.8]
	Let it stand and settle	1.1	[0.2 – 4.4]
	Other	1.8	[0.6 – 5.2]
Stores drinking water		66.2	[44.5 – 82.7]
Storage containers used among households that store drinking water	Jerry can	93.8	[86.5 – 97.3]
	Pot	0.1	[0 – 1.5]
	Bottle	4.5	[2.9 – 7]
	Not available	2	[1 – 4]
	Other	2.7	[1 – 7.4]
Cleaning frequency of container (per week)	7+ times	34.2	[20.8 – 50.6]
	<7 times	60	[45.3 – 73.1]
	Don’t know	5.8	[2.8 – 11.7]
Toilet facility Improved^2^		84.4	[75.5 – 90.4]
Location of toilet facility	In yard or compound	87.4	[81.7 – 91.6]
	Elsewhere	10.8	[7.6 – 15.3]
	Inside the dwelling	1.7	[0.2 – 13.2]
Toilet is clean (no feces/excreta)		51.6	[31.6 – 71.2]
Shares toilet with non-household		18.4	[11.6 – 28]
Type of handwashing facility	No handwashing place	62	[50.5 – 72.3]
	Mobile container (e.g., bucket/jug)	29	[24.9 – 33.5]
	Fixed facility in yard or compound	6.4	[2.7 – 14.2]
	Not permitted to observe	0.2	[0 – 1.5]
	Fixed facility in dwelling (e.g., sink/tap)	2.4	[0.3 – 17.9]
Water available at handwashing site		67.9	[50.8 – 81.2]
Soap available at handwashing site		46.3	[37.7 – 55]

^1^Improved drinking water sources include piped water, protected wells, boreholes, protected springs, rainwater, bottled water, and tanker trucks [22].

^2^Improved toilets include flush to sewer, septic tank, or pit latrine; pit latrine with slab; twin pit with slab; composting toilet; container-based sanitation; and flush to unknown destination [22].

Regarding sanitation, 84.4% of households used an improved toilet. Most toilets were located in the yard or compound (87.4%). About half of households (51.6%) had a toilet that was visibly clean, and nearly one in five (18.4%) shared their toilet with non-household members.

For hand hygiene, mobile containers were the most common type (29%) and 62% of households did not have a designated handwashing place. Overall, 67.9% of households with a handwashing facility had water available, while 46.3% had soap.

## Discussion

This community-based survey assessed MDA coverage for STH and SCH in selected districts of Rwanda, validated reported coverage estimates, explored implementation factors influencing drug uptake, and described WASH conditions. Survey findings confirmed that coverage for both ALB/MBZ and PZQ exceeded the WHO target of 75% and closely matched administrative reports. Perceived receipt of sufficient MDA information to make an informed decision was strongly associated with drug uptake. Nonetheless, important gaps remained, including missed populations and delivery-related challenges such as drug stockouts at some sites during MDA. While access to improved water sources and toilet facilities was relatively high, gaps in toilet cleanliness and handwashing infrastructure persist. Sustaining high MDA participation and achieving long-term control goals will require addressing these delivery challenges and investing in both community engagement and WASH infrastructure.

The high survey-based coverage for ALB/MBZ and PZQ, which exceeded the WHO-recommended threshold and closely aligned with administrative reports, suggests that Rwanda’s MDA monitoring system is functioning effectively. This strong performance is likely attributable in part to the country’s robust community health infrastructure. Trained and experienced CHWs play a critical role in mobilizing households, delivering treatment, and fostering trust through sustained local engagement—contributing to accurate reporting and accountability [24–26]. As a result, Rwanda’s high concordance between survey and reported MDA coverage contrasts with findings from many other settings. For example, a multicounty review reported only 64% overall concordance and 72% among school-aged children between administrative and surveyed MDA coverage [9]. In countries such as Ethiopia, the Democratic Republic of Congo, and Mozambique, administrative coverage has been shown to substantially overestimate true uptake [27,28]. These findings highlight that even in high-performing systems like Rwanda, routine validation surveys remain essential—not only to ensure that programs continue to deliver effectively, but also to maintain data quality, identify underserved subpopulations, and support equitable progress as programs move toward elimination goals.

Despite high overall coverage, specific barriers to both reach and uptake were identified—most notably drug stockouts. Some participants reported that they were not reached because the drugs were unavailable or had run out in their community. These localized stockouts suggest gaps in supply chain management and microplanning, such as underestimation of target population size or delays in drug distribution. Such issues have been widely documented in MDA programs across sub-Saharan Africa, including Kenya and Uganda, where incomplete or delayed deliveries disrupted coverage and contributed to persistent low MDA coverage [29–31]. Strengthening last-mile logistics, improving real-time monitoring during MDA, and ensuring buffer stock availability are critical to preventing these shortfalls.

Findings highlighted the importance of accessibility and interpersonal communication in driving drug uptake. Perception-related factors, particularly perceived receipt of sufficient MDA information to make an informed decision, ease of participation, comfort with the distributor, and comfort with the distribution location, were independently associated with higher uptake. Similar findings from Nigeria and Kenya show that access to information about MDA and engaging an adequate number of distributors to reach the target population are key factors influencing MDA participation [21,30]. Despite the importance of communication in driving uptake, in the unadjusted analysis, individuals who cited brochures, flyers, banners, or social media as information sources were less likely to take the drugs, suggesting that passive communication, while useful for general awareness, may be less effective in building trust to influence drug uptake [32].

One possible explanation is that passive communication channels are more commonly accessed by individuals in urban or peri-urban areas or those with relatively higher socioeconomic status, who may not perceive themselves as targets of NTD programs and may underestimate their personal risk. However, given that only 2.3% of participants reported these as their primary information source, this likely reflects localized patterns rather than a programmatically meaningful association. Nonetheless, it suggests that individuals with higher socioeconomic status or education may require more targeted communication strategies, as evidence from Malawi shows that individuals with tertiary education had three times the odds of non-treatment compared to those with less education [21,30].

This is further supported by our findings that many non-participants gave no specific reason for non-uptake or reported feeling healthy, suggesting low perceived susceptibility. Additionally, some individuals also refused treatment even when offered, citing fear of side effects, mistrust, or uncertainty about the drug. Similar barriers have been reported in Kenya and Ethiopia, particularly among adults [27,30]. These patterns suggest that individuals’ decisions to participate in MDA are influenced by how susceptible they feel to infection and whether they believe the treatment offers clear benefits, particularly in the absence of symptoms [33].

These findings underscore the need to strengthen communication strategies as Rwanda’s NTD program progresses toward elimination. Current MDA messaging may not adequately convey personal risk or the rationale for taking treatment when asymptomatic, limiting its effectiveness among certain groups. Programs should prioritize interpersonal communication through trusted local actors such as CHWs and create opportunities for dialogue to address concerns, and clarify misconceptions. To inform more targeted and equitable approaches, future surveys should include measures of socioeconomic status, such as household assets, education, or housing characteristics, to help explain variations in uptake across population groups.

The study revealed persistent gaps in household WASH conditions. While access to improved water sources and toilets was relatively high, only half of households had visibly clean toilets and 62% lacked a designated handwashing facility. These findings are consistent with national data showing slow progress in WASH coverage [13,14]. More recent evidence indicates that only 72.4% of households use an unshared improved toilet, though this rises to 94.3% when shared facilities are included, consistent with our survey estimates [34]. Limited access to improved and hygienic sanitation likely contributes to sustained STH transmission in Rwanda and may explain why prevalence has declined only marginally in recent years despite consistently high MDA coverage [8,35,36].

These findings align with broader evidence showing that inadequate WASH limits sustained control of STH, even when MDA coverage is high. Improved sanitation and hygiene are well-documented to reduce STH risk [12,37]. In the absence of adequate WASH, reinfection can occur after treatment, limiting the long-term benefits of MDA. Thus, although WASH interventions may have modest short-term impact during ongoing chemotherapy, they remain essential for sustaining progress and ultimately achieving transmission interruption [38]. Embedding WASH promotion—such as handwashing campaigns and improved sanitation—into MDA delivery may therefore enhance effectiveness and sustainability, while routine inclusion of simple WASH indicators such as soap availability and latrine cleanliness in coverage surveys could help identify high-risk households for targeted support.

### Strengths and limitations

This study has several strengths. It used a rigorous, community-based design to generate representative coverage estimates for both STH and SCH. It also collected MDA implementation factors influencing reach and uptake. By validating administrative data and identifying implementation factors for non-reach and non-uptake, the findings provide practical insights to strengthen Rwanda’s MDA program. In addition, the survey collected detailed information on WASH conditions, which are rarely integrated into standard coverage surveys but are critical for sustaining control and elimination.

However, some limitations should be noted. Coverage and uptake were self-reported and may be subject to recall or social desirability bias. Nonetheless, the use of experienced and independent data collectors likely minimized this risk, and prior evidence suggests that individuals can accurately recall drug intake for extended periods, including up to one year [39]. The cross-sectional design also limits causal interpretation of factors associated with uptake. While the study included diverse districts, the purposive selection means findings may not fully represent all contexts in Rwanda. In addition, the survey was not powered to generate district-level estimates, limiting the ability to examine geographic variation in coverage across districts, sectors, and other subgroups. Future evaluations could consider larger, district-representative samples to support sub-national analysis. Finally, while WASH indicators were described, they were not directly linked to drug uptake—an area that could be explored in future studies.

## Conclusion

This study confirms that Rwanda’s MDA program achieved high coverage for both ALB/MBZ and PZQ, consistent with reported administrative data. Uptake was associated with sufficient MDA information, delivery convenience, and comfort with distributors and distribution locations. However, barriers such as drug stockouts and discomfort with delivery settings highlight areas for improvement. While access to improved water and sanitation was relatively high, hygiene gaps—particularly the lack of handwashing facilities—remain a concern and may limit MDA impact. Sustaining high MDA participation and achieving long-term control of STH and SCH will require continued investment in community engagement, addressing operational delivery challenges, and strengthening integration with WASH improvements to maximize program effectiveness and move toward elimination goals.

## Supporting information

S1 Appendix Survey-weighted coverage estimates and unadjusted logistic regression results for ALB/MBZ and PZQ (reach and uptake). (XLSX)
